# *Serratia marcescens* LYGN1 Reforms the Rhizosphere Microbial Community and Promotes Cucumber and Pepper Growth in Plug Seedling Cultivation

**DOI:** 10.3390/plants13050592

**Published:** 2024-02-22

**Authors:** Xu Zhang, Jinxin Peng, Xiaodong Hao, Guifang Feng, Yanhui Shen, Guanghui Wang, Zhiqun Chen

**Affiliations:** 1 College of Life Science, Linyi University, Linyi 276000, China; zhangxu1787@163.com (X.Z.); 13263127966@163.com (J.P.); haoxiaodong@lyu.edu.cn (X.H.); jyxtw@126.com (G.F.); 2 Shandong (Linyi) Institute of Modern Agriculture, Zhejiang University, Linyi 276000, China; shenyh2019@126.com (Y.S.); wgh17806288826@163.com (G.W.)

**Keywords:** *Serratia marcescens*, cucumber, pepper, microbial community, seedlings growth

## Abstract

The vegetable plug seedling plays an important role in improving vegetable production. The process of plug seedling contributes to high-quality vegetable seedlings. The substrate composition and chemical fertilizer are widely studied to promote seedling growth. However, little is known about the effect of beneficial bacteria in the rhizosphere microbial community and vegetables’ growth during plug seedling. The use of beneficial microbes to promote vegetable seedling growth is of great potential. In this study, we showed that the *Serratia marcescens* strain LYGN1 enhanced the growth of cucumber and pepper seedlings in plug seedling cultivation. The treatment with LYGN1 significantly increased the biomass and the growth-related index of cucumber and pepper, improving the seedling quality index. Specifically, LYGN1 also improved the cucumber and pepper root system architecture and increased the root diameter. We applied high-throughput sequencing to analyze the microbial community of the seedlings’ rhizosphere, which showed LYGN1 to significantly change the composition and structure of the cucumber and pepper rhizosphere microbial communities. The correlation analysis showed that the Abditibacteriota and Bdellovibrionota had positive effects on seedling growth. The findings of this study provide evidence for the effects of *Serratia marcescens* LYGN1 on the cucumber and pepper rhizosphere microbial communities, which also promoted seedling quality in plug seedling cultivation.

## 1. Introduction

Vegetable plug seedling could significantly improve seedling quality, having become the widely used way to produce vegetable seedlings [1,2]. Plant growth is often limited by diverse biotic and abiotic stress [3]. For dealing with these environmental pressures in vegetable cultivation, the improvement of plant seedlings quality becomes more and more important. According to the statistics, China produces up to 350 billion plants of professional plug seedlings annually [4], and approximately 60% of the world’s vegetable varieties currently use plug seedling technology [2]. Thus, due to the fact that the demands for vegetable yield and quality are getting higher, researchers are now seeking effective methods to enhance seedling quality.

Cucumber (*Cucumis sativus* L.) is a worldwide cultivated crop which is affected by numerous factors, including temperature, relative humidity, irrigation, fertigation, and disease incidence [5]. Therefore, cucumber plug seedlings are widely used in the improvement of cucumber quality and yield [6]. The plug seedling cultivation of pepper (*Capsicum annuum* L.) also plays a vital role in improving pepper growth and yield after being transformed into soil cultivation [1]. The plant growth-promoting rhizobacteria (PGPR) showed beneficial effects on plant growth [7,8] and also contributed to plant response under different environmental stress [9,10,11]. They can promote plant growth, increase plant biomass, maintain soil fertility, and protect the plant from phytopathogens. PGPR have been increasingly used in vegetable seedling cultivation, proving to improve seeding growth and promote disease inhibition [12]. There still is knowledge gap about the consequences of the application of PGPR on the rhizosphere microbial community and seedling growth in plug seedling cultivation. *Serratia marcescens* is a genetically diverse species which include rhizobacteria with high potential to alleviate biotic stress [13]. The strain of *Serratia marcescens* in this paper was isolated from the cucumber rhizosphere of plants which were grown in a cucumber–amaranth intercropping system [14]. In previous studies, the *Serratia marcescens* NBRI1213 and S-JS1 were respectively observed to promote plant growth and alleviate biotic stress in betelvine [15] and rice [16]. The genomic study of the *Serratia marcescens* strain showed the potential to be used in plant growth promotion or as bioinoculants in agricultural cultivation [17]. Several reports have shown that the application of PGPR, including *Serratia* sp., promotes plant growth and yield of economically important crops [18,19], suggesting the potential of bacteria of this species to improve plant growth in seedling production.

Specific PGPR showed beneficial effects on plant growth, but there is still very little research conducted in plug seedlings cultivation using artificial cultivation substrates. Targeting the requirements for high-quality seedlings in cucumber and pepper cultivation, special attention should be paid to the application of PGPR, which play critical roles in plant growth. This study was conducted to evaluate the effects of LYGN1 on seedling quality, as well as on the cucumber and pepper rhizosphere microbial community in plug seedling cultivation.

## 2. Materials and Methods

### 2.1. Plant Material and Bacterial Strain

Cucumber (*Cucumis sativus* L. cv. ‘Zhongnong 62’) and pepper (*Capsicum annuum* L. cv. ‘Shifeng 802’) were used in the experiment. All seeds were sterilized using 70% ethanol and 3% sodium hypochlorite solution and rinsed with sterile water [20]. *Serratia marcescens* LYGN1 was isolated from the rhizosphere of cucumber in the cucumber–amaranth intercropping system. The strain was deposited at the China General Microbiological Culture Collection Center (CGMCC) under the accession number CGMCC No. 27660.

### 2.2. Experimental Design

The experiment was carried out in a controlled-environment greenhouse at the experimental station of Linyi University, located in the city of Linyi, Shandong Province, China. The cucumbers and peppers were planted in a substrate consisting of peat, vermiculite, and perlite at a ratio of 2:1:1. A single colony was selected in an LB liquid culture medium and incubated overnight at 37 °C to obtain a bacterial suspension. The absorbance of the bacterial suspensions was measured by a spectrophotometer and adjusted to 1 (OD_600_) with a sterile LB medium. The germinated cucumber and pepper seeds were sown in 50-hole trays, which are specifically used for seedling cultivation.

The seedlings of cucumber and pepper were treated with the bacterial suspension of LYGN1 when the cotyledon fully expanded. The bacterial suspension was applied to cucumber and pepper by irrigating it into the root-zone area, with every seedling being irrigated with 20 mL of bacterial suspension [21].

### 2.3. Sampling and Determination of Seedlings Growth

Two time points during the cucumber and pepper seedlings growth were selected. For cucumber, we sampled the cucumber seedlings at 20 and 25 days after inoculation with bacterial suspensions and 44 and 53 days for pepper seedlings. For every treatment, six individual seedlings were sampled to measure the different index. First, the plant height was measured using measuring tape, and the stem thickness was determined by measuring the diameter of the stem base. The total leaf area of peppers was scanned by a scanner (EPSON V800, Seiko Epson Inc., NKS, Tokyo, Japan) and analyzed using software WinRHIZO (LC4800-II LA2400, Sainte Foy, QC, Canada). The cucumber total leaf area was approximated by the product of the measured leaf length, leaf width, and correction factor [22]. The seedlings’ biomass was divided into the shoot biomass and root biomass, which were separately oven-dried at 80 °C until constant weight. The growth rate of cucumber and pepper was calculated by the dividing the change in biomass weight by the number of days for each interval for both cucumber and pepper. Root–shoot ratios were determined by dividing root biomass by shoot biomass. The SPAD chlorophyll values of seedling leaves were measured using a chlorophyll meter, SPAD-502 plus (Konica Minolta, Tokyo, Japan). The seedling vigor index of cucumbers and peppers was calculated according to previous research [23], as follows:

Seedlings vigor index = TDW/(PH/SD + SDW/RDW)

where TDW, SDW and RDW present total dry weight (g), shoot dry weight (g) and root dry weight (g), respectively; and PH and SD are plant height (cm) and stem diameter (mm), respectively.

### 2.4. Root System Architecture Analysis

For the cucumber and pepper root architecture analysis, the cucumber and pepper seedlings at two time points at the end of the experimental trial were carefully extracted from the substrate, scanned using a scanner (EPSONV 800), and analyzed by software WinRHIZO. For each species, the following root architecture measurements were measured: total root length, the number of root tips, average root diameter, and the total root surface area. 

### 2.5. Microbial Community of Cucumber and Pepper Rhizosphere 

The rhizosphere microbial community genomic DNA of cucumber and pepper was extracted and purified from about 400 mg substrate using the PowerSoil DNA isolation kit (QIAGEN Inc., Santa Clarita, CA, USA). The primers 515F (5′-GTGCCAGCMGCCGCGGTAA-3′) and the reverse primer 806R (5′-GGACTACHVGGGTWTCTAAT-3′) [17,24], as well as the primers ITS1F (5′-CTTGGTCATTTAGAGGAAGTAA-3′) and the reverse primer ITS2R (5′-GCTGCGTTCTTCATCGATGC-3′) were used to amplify the 292 bp fragment of the V4 region in the 16S rRNA gene and for the amplification of the 300 bp fragment of the fungal ITS gene [18,25], respectively. PCR was performed under the following conditions: 95 °C for 3 min, followed by 27 cycles at 95 °C for 30 s, 55 °C for 30 s and 72 °C for 45 s, with a final extension at 72 °C for 10 min. The sample libraries for sequencing were prepared according to the MiSeq Reagent Kit Preparation Guide (Illumina, Inc., San Diego, CA, USA) and sequenced using the Illumina MiSeq platform. The high-throughput sequencing was performed by Majorbio (Shanghai Majorbio Bio-pharm Technology Co., Ltd., Shanghai, China).

Illumina sequences of 16S and the ITS were processed and sequentially quality-filtered using Fastp (version 0.19.6) [26]. Pair-end reads were merged with a minimum overlap using Flash (1.2.11) [27]. After removing chimeric sequences, the remaining sequences were binned into operational taxonomic unit (OTU) with 97% similarity and the representative sequence for each OTU was taxonomically classified via the Ribosomal Database Project’s classifier [28], the SILVA database (version 138) [29] for bacteria, and the UNITE (version 8.0) for fungi [30]. All OTUs identified as belonging to chloroplast and mitochondria were removed from the data set. Then, the representative sequences for each OTU were aligned using PyNAST [31] in QIIME [32] and carried out by Uparse software (version 11) [33].

### 2.6. Statistical Analysis

The data of seedlings growth were subjected to analysis using Student’ *t*-test, carried out by SPSS 26.0. To avoid the bias caused by different sequencing depths, the least number of obtained sequences from all microbial samples was used for normalization. The Shannon index, Chao1, and the Simpson index were applied to directly compare the α-diversity of the root-zone soil microbial community. Non-metric multidimensional scaling was used to assess the β-diversity of the microbial community. The rhizosphere microbial community data were analyzed using the online platform Majorbio Cloud Platform (www.majorbio.com, accessed on 19 January 2023). For correlation analysis, Pearson product–moment correlation coefficient (Pearson’s r) analysis was performed. The raw sequencing data of the bacteria and fungi were submitted into the NCBI Sequence Read Archive (SRA) database (Accession Number: PRJNA1051178).

## 3. Results

### 3.1. The Cucumber and Pepper Seedling Traits and Root Morphology

**Figure 1:**
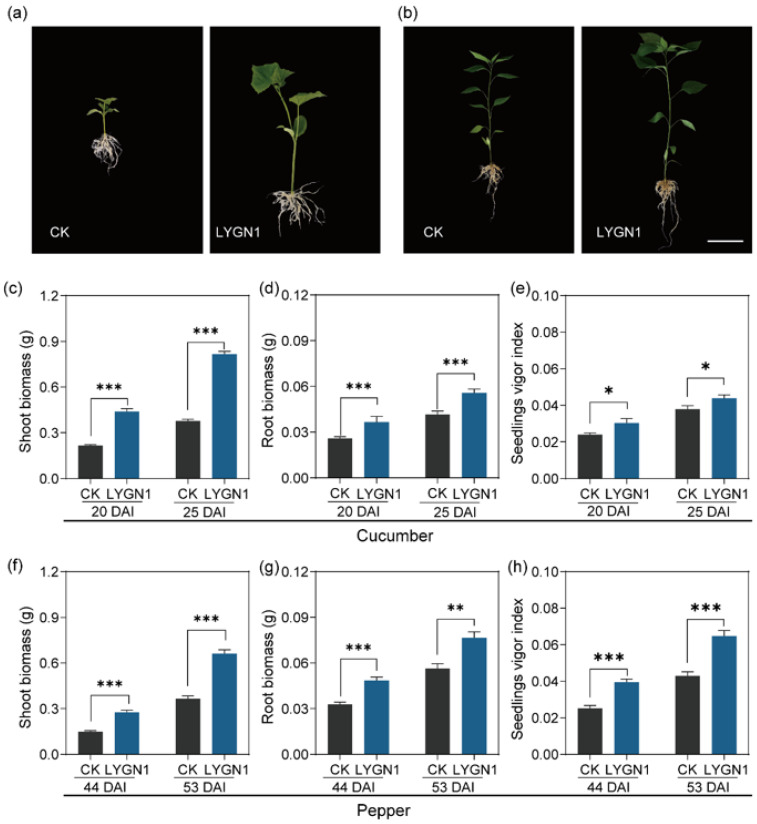
Effects of LYGN1 treatment on cucumber and pepper biomass accumulation and seedlings vigor index. CK: control; LYGN1: cucumber and pepper seedlings treated with *Serratia marcescens* LYGN1; DAI: days after inoculation. Representative pictures of cucumber (**a**) and pepper (**b**) seedlings treated with control and LYGN1, scale bar: 5 cm. The biomass of shoot (**c**) and root (**d**), and the seedlings vigor index (**e**) in cucumber seedlings. The biomass of shoot (**f**) and root (**g**), and the seedlings vigor index (**h**) in pepper seedlings. Data are shown as mean ± SEM, *p*-values, calculated using Student’ *t*-test, * *p* < 0.05, ** *p* < 0.01, *** *p* < 0.001, *n* = 6.

**Figure 2:**
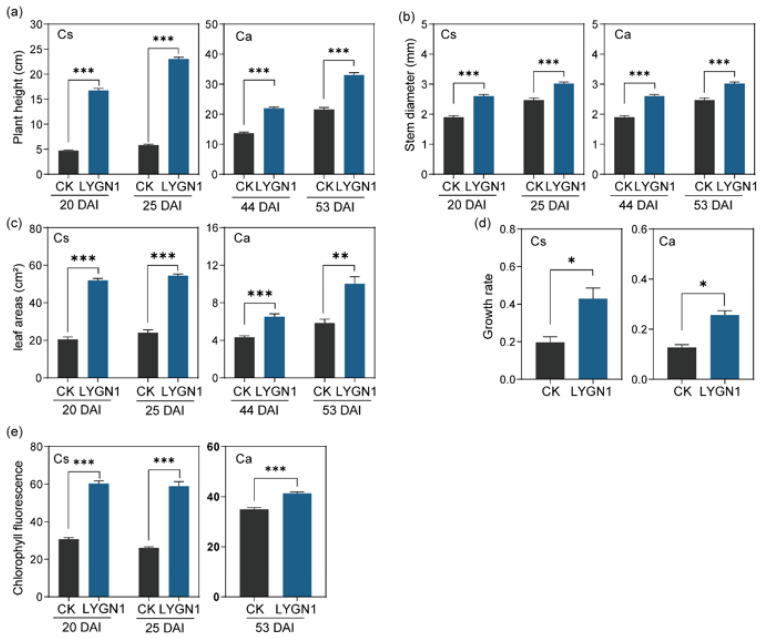
Effects of LYGN1 treatment on growth-related indexes of cucumber and pepper seedlings. CK: control; LYGN1: cucumber and pepper seedlings treated with *Serratia marcescens* LYGN1; DAI: days after inoculation; Cs: *Cucumis sativus*; Ca: *Capsicum annuum*. (**a**) The plant height of cucumber and pepper seedlings at different sampled timepoints. (**b**) The stem diameter of cucumber and pepper seedlings at different sampled timepoints. (**c**) The leaf area of cucumber and pepper seedlings at different sampled timepoints. (**d**) The growth rate of cucumber and pepper seedlings at different sampled timepoints. (**e**) The Chlorophyll fluorescence of cucumber and pepper seedlings at different sampled timepoints. Data are shown as mean ± SEM, *p*-values, calculated using Student’ *t*-test, * *p* < 0.05, ** *p* < 0.01, *** *p* < 0.001, *n* = 6.

The application of LYGN1 had the same trends in the biomass accumulation of cucumber and pepper. The shoot and root biomass of cucumber were significantly increased after being inoculated with the bacterial suspensions (*p* < 0.001) (Figure 1). In this study, the shoot and root were sampled at two timepoints following the cucumber and pepper growth. Positive effects of LYGN1 application on seedling biomass accumulation were observed at two sampled timepoints (Figure 1). As showed in the representative picture of cucumber and pepper seedlings at 25 and 53 DAI, respectively, the seedlings treated with LYGN1 were obviously larger than the control (CK) treatments (Figure 1a,b). We also measured several growth indexes in cucumber and pepper plants to evaluate the effects of LYGN1 application. Through many plant traits, we demonstrated that the effect of plant growth promotion was strongly induced in the LYGN1 treatment group (Figure 2). The plant height and stem diameter were both significantly improved under LYGN1 treatment, which showed a great promoting effect. Meanwhile, the growth rate of cucumber and pepper seedlings was significantly higher in the LYGN1-treated plants, which also had the higher chlorophyll fluorescence (SPAD). Taken together, LYGN1 treatment enhanced biomass accumulation and promoted seedling growth.

**Figure 3:**
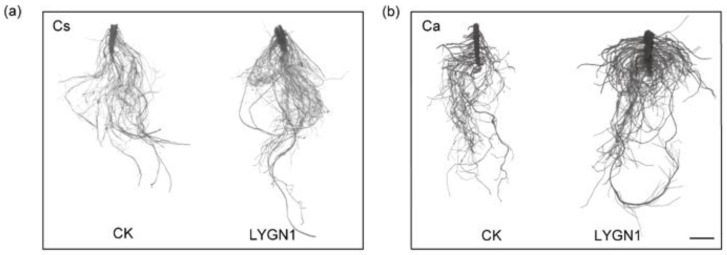

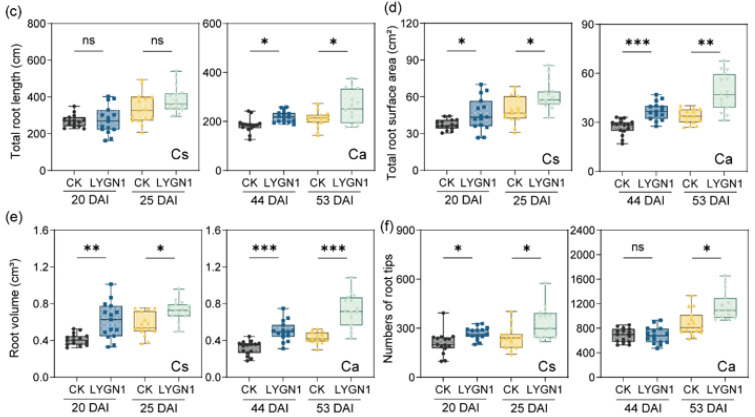
Effects of LYGN1 treatment on root system growth of cucumber and pepper seedlings. CK: control; LYGN1: cucumber and pepper seedlings treated with *Serratia marcescens* LYGN1; DAI: days after inoculation; Cs: *Cucumis sativus*; Ca: *Capsicum annuum*. Representative pictures of cucumber (**a**) and pepper (**b**) root system treated with control and LYGN1, scale bar: 5 cm. (**c**) The total root length of cucumber and pepper seedlings. (**d**) The total root surface area of cucumber and pepper seedlings. (**e**) The root volume of cucumber and pepper seedlings. (**f**) The number of root tips of cucumber and pepper seedlings. The *p*-values were calculated using Student’ *t*-test, * *p* < 0.05, ** *p* < 0.01, *** *p* < 0.001, ns: no significance, *n* = 15.

**Figure 4:**
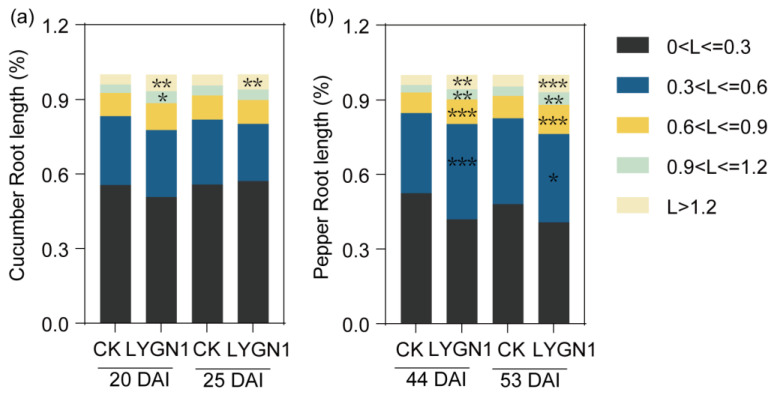
Effects of LYGN1 treatment on root system architecture. CK: control; LYGN1: cucumber and pepper seedlings treated with *Serratia marcescens* LYGN1; DAI: days after inoculation. The roots were divided into five groups, according to their diameters (L, mm). (**a**) The root classification of cucumber seedlings’ root system. (**b**) The root classification of the pepper root system. The *p*-values between the control and LYGN1 treatment were calculated using Student’ *t*-test, * *p* < 0.05, ** *p* < 0.01, *** *p* < 0.001, *n* = 15.

All the parameters measured and calculated by WinRHIZO system software are shown in Figure 3. The root system of cucumber (Cs) and pepper (Ca) seedlings was larger and had more fine roots (Figure 3a,b). Especially for the pepper seedlings, the total root length was significantly higher in the LYGN1 treatment (S) at both sampling timepoints (Figure 3c). Another important component of a functional root system is the root surface area, which represents the total area of the root system that is in contact with the substrate. LYGN1 treatment significantly increased root surface, especially for the pepper root at 44 DAI (Figure 3d). In this study, LYGN1 could also affect root volume and root tip development. Overall, both cucumber and pepper seedlings showed a significant promoting effect (Figure 3f). Based on the diameter of the root, roots were categorized in five grades (Figure 4). LYGN1 application facilitated the growth of many more lateral roots, with thicker diameter, in the cucumber and pepper seedlings (Figure 4). Especially during the growth of pepper seedlings, this phenomenon was more significant (Figure 4b). In short, the application of LYGN1 significantly promoted the growth of cucumber and pepper seedlings.

### 3.2. Microbial Community Diversity of Cucumber and Pepper Rhizosphere

**Figure 5:**
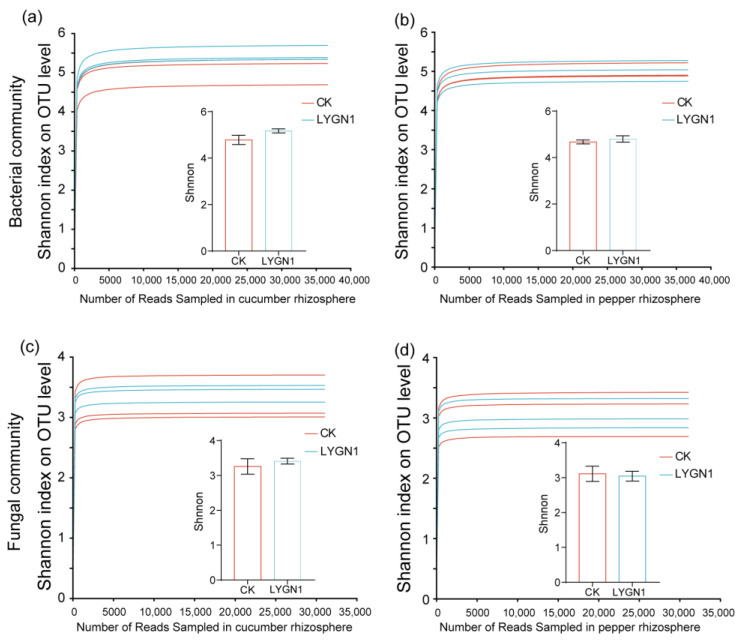
Effects of LYGN1 treatment on α-diversity of cucumber and pepper rhizosphere microbial community. CK: control; LYGN1: cucumber and pepper seedlings treated with *Serratia marcescens* LYGN1; OUT: operational taxonomic unit. The Shannon index of the bacterial community in cucumber (**a**) and pepper (**b**) under LYGN1 treatment. The Shannon index of the fungal community in cucumber (**c**) and pepper (**d**) under LYGN1 treatment. Data are shown as mean ± SEM, *n* = 3.

**Figure 6:**
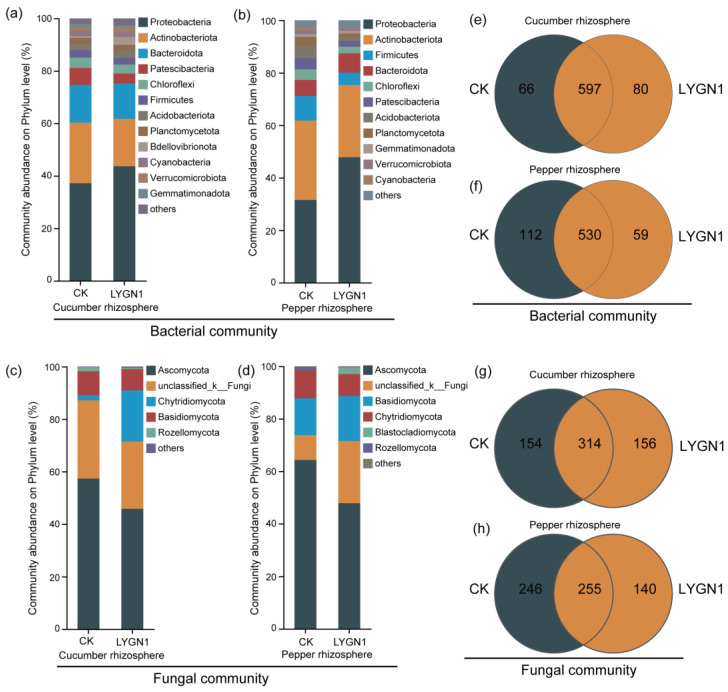
Effects of LYGN1 treatment on the composition of cucumber and pepper rhizosphere microbial communities. CK: control; LYGN1: cucumber and pepper seedlings treated with *Serratia marcescens* LYGN1. The relative abundance of cucumber (**a**) and pepper (**b**) rhizosphere bacterial communities at phylum level. The Venn diagram shows the numbers of specific and shared OTUs between the cucumber (**e**) and pepper (**f**) rhizospheres under LYGN1 treatments. The relative abundance of cucumber (**c**) and pepper (**d**) rhizosphere microbial communities at phylum level. The Venn diagram shows the numbers of specific and shared OTUs between cucumber (**g**) and pepper (**h**) rhizosphere microbial communities after LYGN1 treatment.

**Figure 7:**
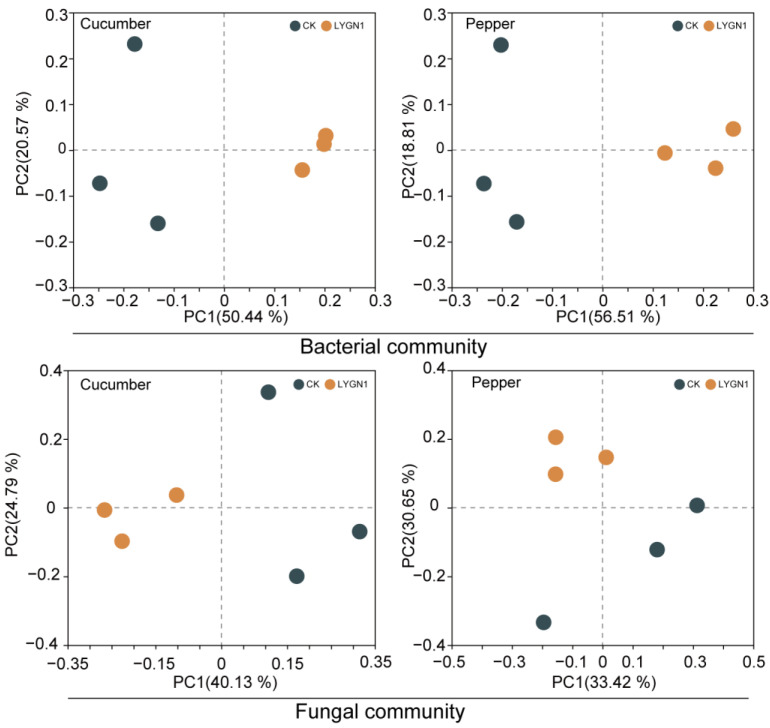
Effects of LYGN1 treatment on the β-diversity of cucumber and pepper rhizosphere microbial communities. Principal coordinate analysis (PCoA) was based on Bray–Curtis distances. CK: control; LYGN1: cucumber and pepper seedlings treated with *Serratia marcescens* LYGN1.

The rhizosphere microbial community is known to greatly promote plant growth. We also investigated the effects of the application of LYGN1 on cucumber and pepper rhizosphere microbial community. The smallest numbers of bacterial sequences (33,820 sequences per sample) and fungi (31,098 sequences per sample) were normalized to avoid the deviation caused by the effects of different sequencing depths. A total of 2,776,766 raw reads were obtained for all the samples described in this study. After filtering, 2,224,310 reads and 4900 OTUs were obtained. The Shannon index of cucumber at the OTU level was higher after the LYGN1 application compared to the control. Both bacterial and fungal communities showed this trend in cucumber. However, the application of LYGN1 slightly decreased the Shannon index of fungal communities in pepper seedlings (Figure 5). These results showed the different response of LYGN1 application in cucumber and pepper. In the bacterial community, the relative abundance of Proteobacteria was significantly higher in the LYGN1 treatment in both cucumber and pepper rhizospheres (Figure 6a). Especially in the pepper rhizosphere, the relative abundance of *Proteobacteria* increased 16.29% in the LYGN1 treatment compared to the control after 53 DAI. The relative abundance of Bacteroidota in the rhizosphere of cucumber and pepper showed an opposite trend, with an increasing trend in pepper (Figure 6a,b). The addition of LYGN1 had a greater impact on Ascomycota in the fungal community, reducing its relative abundance in the rhizosphere of cucumber and pepper (Figure 6c,d). These results showed that *Serratia marcescens* may affect the colonization of Ascomycota on the cucumber and pepper rhizospheres. The Venn diagrams show the shared and unique OTUs in the bacterial and fungal community (Figure 6e,g), which exhibited different effects on the cucumber and pepper microbial communities. The principal coordinates analysis (PCoA) of the bacterial and fungal communities also showed the significant separation between LYGN1 treatment and control conditions in both cucumber and pepper rhizospheres (Figure 7), demonstrating the effect of LYGN1 application on the microbial communities of cucumber and pepper seedling rhizospheres.

### 3.3. Correlation Analysis of Phylum-Level Microbial Groups and Seedlings Index

**Figure 8:**
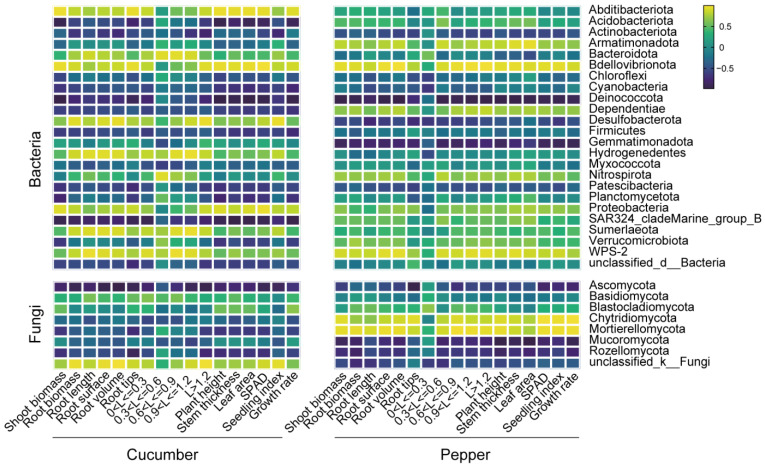
Correlation analysis between the phylum-level microbial groups and the seedling growth index. The heatmap shows the positive and negative correlations between the changes in phylum-level microbial groups and different seedlings index. Pearson’s product–moment correlation coefficient (Pearson’s r) analysis was performed. The data used for the analysis of all samples were taken from the latest sampling period. The seedlings growth-related index includes shoot and root biomass, stem thickness, leaf area, SPAD (chlorophyll fluorescence), seedling index, and growth rate. The root system-related index includes root length, root surface area, root volume, and root tips, as well as the five group of roots classified according to their diameters (L, mm).

We measured the different plant growth and root system index of cucumber and pepper seedlings, and the correlation analysis revealed the association between the seedlings and annotated microbial community at phylum level (Figure 8). The *Abditibacteriota* significantly affect the cucumber growth positively, especially for the shoot biomass and seedlings growth rate (Figure 8) but affect pepper growth slightly. The relative abundance of the phylum *Chloroflexi*, *Cyanobacteria*, *Deinococcota,* and *Dependentiae* was significantly decreased in cucumber rhizosphere but showed negative effects on cucumber shoot and root growth. The changes in fungal community caused different effects on the cucumber and pepper indicators (Figure 8). Pearson’s correlation analysis showed significant positive correlations between *Ascomycota* and seedling indicators, but in the cucumber and pepper rhizosphere, the phylum *Chytridiomycota* and *Mortierellomycota* showed opposite correlations with the different indictors. All these results showed the effects of the LYGN1 application on microbial communities and the subsequent effects on cucumber and pepper seedling growth.

## 4. Discussion

Previous studies showed that the application of PGPR could promote plant growth by reforming the rhizosphere microbial environment [6,34]. In this study, we showed that LYGN1 treatment has the same trends. The structure of the bacterial and fungal communities in both cucumber and pepper rhizosphere was reformed. Meanwhile, the application of LYGN1 in cucumber and pepper plug seedlings showed significant promotion effect on both seedling growth and root development. This promotion effect showed the same trends in the two sampled timepoints after the LYGN1 treatments.

The compact seedlings were demonstrated to lead to healthier plants, higher yields, and improved nutritional qualities compared to weak seedlings after transplanting [35]. Therefore, the cultivation of high-quality vegetable seedlings is vital for increasing crop yield and farmer income. In our study, the seedling vigor index was significantly higher in the LYGN1 treatment, showing its potential in cucumber and pepper seedling cultivation. Plant growth-promoting rhizobacteria (PGPR) can promote biomass accumulation and plant growth [36]. This finding is the same as that in our study, which demonstrated that the application of LYGN1 could significantly promote the growth of cucumber and pepper. Studies showed that *S. marcescens* can promote the growth of various plants by producing IAA [17,19,37], which can also significantly increase root growth. The chlorophyll fluorescence in this study had a significant increase after the LYGN1 application, so the enhanced chlorophyll fluorescence parameters may have contributed to better plant growth during the PGPR treatment [38]. The growth and development of seedlings above the ground are significantly related to the root system. In this study, the root biomass was significantly increased in both cucumber and pepper. Furthermore, marked differences among the control and LYGN1 treatment were found in root architecture. Beneficial microbes could affect root development, and the altered root system architecture may improve seedling growth by nutrient accumulation [39]. The total root surface area contributes to an increase in the total absorptive surface of the root system [40]. Additionally, total length and root volume could also promote the absorption of more available nutrients by cucumber and pepper seedlings. We also conducted a root diameter classification analysis by selecting a range of root diameters. The results showed that LYGN1 significantly increased the root diameter, resulting in thicker roots, especially in pepper seedlings, which may show a longer lifespan and enhanced function [41]. All these indicators show the great potential of LYGN1 to promote cucumber and pepper seedling growth and root development. PGPR have been widely studied for improving plant growth and productivity [8,42], and the rhizosphere microbial community plays important roles in promoting plant growth and in improving tolerance to disease and abiotic stress [43]. However, there are few studies evaluating the rhizosphere microbial communities, especially under PGPR treatment, in plug seedling cultivation. *Serratia marcescens* was demonstrated to induce plant defense and improve significant growth increases in shoot length, shoot dry weight, root length, and root dry weight [15]. Additionally, it also showed its varied beneficial traits and plant growth-promoting potential in coconut palms [19]. In this study, the α-diversity was slightly increased after the *Serratia marcescens* LYGN1 application, but *Serratia marcescens* LYGN1 significantly changed the structure of both bacterial and fungal communities. The obtained results from Pearson’s correlation analysis showed the specific phylum which is related to seedling growth. The correlation analysis showed that the Abditibacteriota and Bdellovibrionota had positive effects on seedling growth. The relative abundance of Proteobacteria were increased in both cucumber and pepper. Previous studies reported that Proteobacteria were the dominant taxonomic phyla in lettuce substrate seedlings, which is also related to the promoting effects on lettuce growth [44]. The Proteobacteria phyla also comprise several PGPR species that promote plant growth [45], and the changes were more intense in the pepper rhizosphere, which may contribute to the growth. Furthermore, Proteobacteria also showed positive correlations with different seedling growth and root system indicators. The Firmicutes taxa have the potential to enhance plant stress tolerance, growth, and nutrient uptake [46]. However, the Firmicutes were decreased in both cucumber and pepper rhizospheres, which also showed negative correlations with different seedling growth and root system indicators. The relative expression of Ascomycota in cucumber and pepper was decreased and showed negative correlations with the related indicators. Previous studies showed that the initial colonization by PGPR in the rhizosphere affected the microbial community composition throughout the plant growth stages [47], which showed the potential of PGPR inoculation during seedling cultivation for promoting vegetable growth and yield.

## 5. Conclusions

In this study, the *Serratia marcescens* LYGN1 was used for its growth-promoting effects in plug seedling cultivation. LYGN1 improved root growth and the seedling vigor index, significantly promoting cucumber and pepper seedling growth. Our results also reveal that the microbial community composition of cucumber and pepper rhizospheres was reformed. Additionally, the correlation analysis showed that the changed microbial groups could affect seedling growth and root system development. These results show the great potential of LYGN1 in cucumber and pepper plug seedling cultivation, which could improve the growth and yield in subsequent soil cultivation. In further studies, it will also be interesting to profile the metabolites to understand the mechanism involved.

## Data Availability

The raw data for 16S rRNA gene and ITS sequences have been submitted to the NCBI Sequence Read Archive (SRA) database under the BioProject number PRJNA1051178.
